# One size does not fit all: a qualitative exploration of the experiences of Canadian youth with neurodevelopmental disabilities during the COVID-19 pandemic

**DOI:** 10.3389/fpubh.2025.1637153

**Published:** 2025-10-13

**Authors:** Brittany Finlay, Ashish Seth, Genevieve Currie, Christiane Roth, Anne Hudon, Matthew Hunt, Lucyna M. Lach, David B. Nicholas, Keiko Shikako, Jennifer D. Zwicker

**Affiliations:** ^1^School of Public Policy, University of Calgary, Calgary, AB, Canada; ^2^School of Nursing and Midwifery, Mount Royal University, Calgary, AB, Canada; ^3^School of Rehabilitation, Faculty of Medicine, Université deMontréal, Montreal, QC, Canada; ^4^School of Physical and Occupational Therapy, McGill University, Montreal, QC, Canada; ^5^School of Social Work, McGill University, Montreal, QC, Canada; ^6^Faculty of Social Work, University of Calgary, Calgary, AB, Canada; ^7^Faculty of Kinesiology, University of Calgary, Calgary, AB, Canada

**Keywords:** youth, neurodevelopmental disability, COVID-19, mental health, services, disability-inclusive policy, co-design

## Abstract

**Introduction:**

Youth with neurodevelopmental disabilities (NDD) were disproportionately impacted by the COVID-19 pandemic due to health and socioeconomic factors and system level disruption of essential supports. To date, few studies have engaged directly with youth with NDD to understand how they were been impacted by the pandemic. The aim of this study was to uncover experiences of youth with NDD during the COVID-19 pandemic.

**Methods:**

Purposive sampling was used to recruit Canadian youth (age 18–30, inclusive) with NDD. Participants were provided with the option of participating in a written (online) or verbal (Zoom) interview. Deductive coding and inductive analysis were used to develop themes.

**Results:**

Forty youth participated in an interview. We discuss the impacts of the COVID-19 pandemic on participants in five key areas: education and academic performance, access to disability and healthcare services, social connectedness, participation in society, and mental health. Within these areas, both positive and negative experiences were reported. A secondary finding emerged related to the impact of gender identity on access to services.

**Discussion:**

Our study highlights the need for policy approaches that are flexible and responsive to the variability of needs among Canadian youth with NDD moving forward.

## 1 Introduction

The COVID-19 pandemic was an unprecedented time in history that impacted the health and well-being of people worldwide. Due to pre-existing inequities in institutional and social structures, certain groups were especially vulnerable to the impacts of the COVID-19 pandemic (1). One such group is adolescents and young adults (referred to as youth, hereafter) with neurodevelopmental disabilities (NDD). NDDs are a group of long-term conditions originating in the brain and/or neuromuscular system that are associated with functional limitations (2). The majority of youth with disability in Canada live with a NDD (estimated 75 percent) (3).

Multiple factors contributed to the disproportionate impacts of the COVID-19 pandemic for youth with NDDs and their families. Many youths with NDD have frequent contact with a number of people who provide support, have other associated health conditions, and had difficulties adhering to precautionary health measures, which, in combination, increased the risk of a COVID-19 infection and associated adverse health outcomes (4, 5). Additionally, youth with NDD and their families faced disproportionate economic and mental health impacts during the pandemic, due to overrepresentation in lower socioeconomic groups and higher prevalence of pre-existing mental health issues (4, 6).

While other studies have looked at impacts of the COVID-19 pandemic on children and youth with disabilities, the majority of the literature relied on proxy perspectives provided by parents and caregivers and focused primarily on younger (i.e., school-aged) children (7–21). Few studies to date have engaged with youth with NDDs directly to understand and learn from their experiences during the COVID-19 pandemic as they transition into adulthood. The developmental transition from childhood to adulthood involves many life changes and, for youths with NDD, often involves the loss of children- and family-oriented social services and supports provided in a school setting (22), making this life stage an important area for exploration. Additionally, most of the literature published to date utilized quantitative approaches, such as surveys or questionnaires, to determine the impacts of the COVID-19 pandemic on youth and their families. While these approaches provide important data and valuable contributions to the literature, they can be enriched by a nuanced, comprehensive view that youth themselves can provide of the impacts of the COVID-19 pandemic on different aspects of their life.

In this paper, we address these gaps in the literature using a qualitative approach to provide a comprehensive overview of the impacts of the COVID-19 pandemic and associated precautionary health policies from the perspective of Canadian youth with NDDs. Specifically, our objective was to identify how the COVID-19 pandemic affected different aspects of the lives of youth, such as access to services, education, employment, mental health, and activities of daily living. An in-depth understanding and learning from youths’ experiences during the COVID-19 pandemic is critical for understanding how to ensure the needs of youth with NDD are met when planning for future emergency situations, and, more broadly, to move towards more responsive and tailored supports systems.

## 2 Materials and methods

### 2.1 Study design

This study used a qualitative research design using Interpretative Description (ID), which allows for a nuanced exploration of health and human experience from a holistic perspective that considers complex interactions between psychosocial, biological, and relational phenomena (23). Our approach used interviews to better understand the experiences of youth with NDD and their families during the COVID-19 pandemic. The design and methodology of this study was created in partnership with our stakeholder advisory council, which consisted of youth with disabilities, parents and caregivers of youth with disabilities, and disability advocates. We also collaborated with members of the CHILD-BRIGHT National Youth Advisory Panel (NYAP) (24) for specific guidance on data collection and recruitment methods to ensure accessibility of the study for youth with NDDs. This study was approved by the University of Calgary Conjoint Faculties Research Ethics Board (ID: REB17-1585).

### 2.2 Participant sample and recruitment

Participant inclusion criteria included youth aged 18–30 years (inclusive), residing in Canada self-identifying as having at least one NDD. We selected a lower bound of 18 years to ensure individuals were able to provide their own consent and participate in the study independently of their parents or caregivers, if desired. This was to ensure that participants were able to provide information that reflected their own experiences, and not that of their parents or caregivers. We selected 30 years as the upper bound to ensure we captured information across different stages of early adulthood (i.e., transition from child support system into adult support system, entering and exiting post-secondary education, and entering the labour force). Participants were also required to speak either English or French.

To assess eligibility for study inclusion and collect sociodemographic information from potential participants, an online screening survey was developed using the University of Calgary Clinical Research Unit Research Electronic Data Capture (REDCap) platform. At the end of this survey, participants were asked to indicate whether they were interested in participating in an interview to discuss their experiences during the COVID-19 pandemic, and whether they preferred to participate in a written or verbal interview (described in further detail below).

A convenience and purposeful sampling approach was utilized between June and August 2021, whereby the link to the online screening survey was shared with the research team, individuals that have previously participated in studies with our research groups, advisory council members, and through research networks where researchers were affiliated. We also shared the survey link on various social media platforms, such as Facebook, Instagram, and X (formerly known as Twitter). To obtain a geographically diverse sample, Facebook and Instagram ads targeting specific regions in Canada were used, as a means of purposive sampling.

Our primary goal in selecting participants for the interviews was to achieve maximum geographic representation across Canadian provinces, by completing at least two interviews per province. For provinces with a smaller pool of potential participants (less than five individuals), we contacted all individuals to request participation in a follow-up interview. For provinces with a larger pool of potential participants (i.e., from provinces where the researchers have stronger networks, and provinces with larger populations), we utilized other sociodemographic characteristics (such as income level, community type, and gender) to guide recruitment decisions so as to obtain a diverse sample, respecting a purposeful and maximum variation criteria.

### 2.3 Data collection

Youth that completed the screening survey and that were selected to participate in the interview had the option to complete a verbal or written interview in either English or French. Interviews were completed between June and August 2021. Participants who completed the verbal or written interview were provided with a $25 e-gift card.

Originally, our team planned to exclusively conduct verbal interviews over Zoom, to mirror our methods in a concurrent, complimentary study investigating the experiences of parents and caregivers of youth with NDD during the COVID-19 pandemic (9, 19). However, our stakeholder advisory council and youth research partners expressed concerns that a verbal interview over Zoom may not be accessible to and inclusive of all youth with NDD, prompting us to include an written online interview option. This written online option provided an opportunity for our team to reach youth that may not be able to participate in a verbal interview for various reasons relating to their disability (for example, lack of verbal communication abilities, co-occurring social anxiety, etc.). Additionally, this option allowed youth to complete the interview at their own pace and at a time that was convenient for them.

Verbal interviews were conducted using Zoom and were semi-structured. The interview guide developed for the aforementioned parent/caregiver study was used for this study, but language was modified to ensure its applicability to the youth cohort. Language in the parent/caregiver interview guide was modified to refer to the youth participant completing the interview instead of the child of the parent/caregiver. A detailed overview of the methods for creating the interview guide is provided elsewhere (9, 19). Consent was obtained through a verbal consent process, whereby interview participants verbally confirmed that they had reviewed the consent form, understood the information in the consent form, and agreed to participate. Interviews were audio recorded through the Zoom platform.

The written interview included open-ended and multiple-choice questions to capture the same information as the verbal interviews. The written interview was hosted through the REDCap platform. Consent was obtained through an implied consent process, whereby participants were required to review the consent form embedded in the written interview. To ensure that participants understood the information provided in the consent form and agreed to participate in the study, they were required to answer “yes” to four mandatory consent-related questions. Answering “no” to any of these questions would trigger the online interview to automatically close and end their participation. All questions in the written interview, with the exception of the consent questions, were optional for participants to answer, to ensure that participants only answered questions that they were comfortable answering. In an effort to mimic the semi-structured nature of the verbal interviews that allows for probing, we noted in the consent form that we may follow-up with participants via email with the purpose of further clarifying or expanding on their written responses in the interview. This also allowed us to follow up with participants in the event of a large amount of missing data from their written interview.

### 2.4 Data analysis

Descriptive analyses of screening survey data were completed using Microsoft Excel. For the purposes of this paper which focuses on a qualitative analysis, we only utilized the open-ended data from the written interview. These data were compiled into a single document for each participant and analyzed in an identical manner to a verbal interview transcript. French open-ended data were translated into English for analysis. Interview recordings were transcribed verbatim using REV transcription software. Transcript accuracy was validated by a member of the research team (BF).

Verbal interview transcripts and written, open-ended data were coded in NVivo 12. The initial codebook was created using a deductive approach, whereby codes were developed based on the interview guide to organize interview data into categories and sub-categories (25). These categories were as follows: Access to Services, Employment and Education Impacts, Gender and Services, Information about COVID-19, Mental Health, Other Personal Changes as a Result of COVID-19, Precautionary Health Measures, and Vaccine. Each category and sub-category had a definition and specific inclusion and exclusion criteria to ensure consistency across coders and establish rigor in the coding process. The three coders (BF, AS, GC) each coded the same three transcripts independently using the initial codebook, then met virtually to compare coding and assess whether the codebook required any additional or revised categories or codes. Following this meeting, the remaining transcripts and open-ended data were split between the three coders, and all coding was merged to facilitate analysis across participants. Following this initial phase where the data were sorted into categories, inductive coding was completed within each category (25), allowing coders to extract key messages from each category. Finally, inductive analysis by the research team (BF, GC, AS, CR, JZ) within and across categories was utilized to develop themes from the key messages. This involved looking for patterns among the key messages, grouping similar key messages into potential themes, and validating themes by reviewing and evaluating them to ensure they accurately represented the collected data. Themes were also reviewed and evaluated by the broader research team and stakeholder advisory council to ensure they adequately reflected the study findings.

## 3 Results

### 3.1 Participant characteristics

During the recruitment period, 144 individuals completed the online screening survey. Among those interested in participating in an interview (*N* = 61), 40 individuals completed an interview. Two individuals had previously participated in research with our team; the remaining 38 were recruited via social media, referrals from other participants, and connections of the research team and advisory council. Sociodemographic characteristics of participants are described in Table 1. The majority of participants completed the interview in English (90.0%) and online (62.5%). Our sample consisted of individuals residing across Canada, with representation from eight provinces, and residing in various community types, with the majority residing in large urban centres (57.5%). Our sample exhibited significant diversity with respect to educational attainment and gender identity. Notably, 27.5% of our sample did not identify with the male–female gender binary. The majority of our sample consisted of individuals identifying as non-Indigenous (85.0%) and with a total household income of less than $40,000 (60.0%).

**Table 1:** Participant sociodemographic characteristics (*N* = 40).

Characteristic	Number of participants	Percentage
Language of interview		
English	36	90.0%
French	4	10.0%
Interview type		
Written (online)	25	62.5%
Verbal (Zoom)	15	37.5%
Gender		
Male	8	20.0%
Female	20	50.0%
Other (including non-binary, genderfluid, two-spirit, etc.)	11	27.5%
Educational attainment		
Some High School	6	15.0%
High School Graduation	5	12.5%
Some College/Technical Training	6	15.0%
College/Technical Training Grad	3	7.5%
Some University	11	27.5%
University Graduate	9	22.5%
Indigenous self-identification		
First Nations Status	2	5.0%
First Nations Non-Status	1	2.5%
Métis	3	7.5%
Non-indigenous	34	85.0%
Population centre		
Large Urban (≥100,000 people)	23	57.5%
Medium (30,000–99,999 people)	4	10.0%
Small (1,000–29,999 people)	6	15.0%
Rural (<1,000 people)	2	5.0%
Province of residence		
Alberta	6	15.0%
British Columbia	6	15.0%
Manitoba	3	7.5%
New Brunswick	2	5.0%
Nova Scotia	7	17.5%
Ontario	8	20.0%
Quebec	6	15.0%
Saskatchewan	2	5.0%
Total household income (2019)		
<$40,000	24	60.0%
$40,000–$79,999	7	17.5%
>$80,000	2	5.0%

Participant responses for sociodemographic questions were excluded in this table if the participant did not select a response or if they selected “prefer not to answer” or “do not know.” The number of respondents for some characteristics may not equal the total number of participants, and percentages may not add up to 100 percent.

Disability characteristics of participants are described in Table 2. The majority of our sample comprised individuals with one NDD diagnosis (67.5%) and with a previous mental health diagnosis (85.0%). The most common diagnosis in our sample was autism spectrum disorder (52.5%).

**Table 2:** Participant disability characteristics (*N* = 40).

Characteristic	Number of participants	Percentage
Primary NDD Diagnosis^*^		
Autism Spectrum Disorder (ASD)	21	52.5%
Attention Deficit Hyperactivity Disorder (ADHD)	9	22.5%
Cerebral Palsy	4	10.0%
Tourette’s Syndrome	3	7.5%
Fetal Alcohol Spectrum Disorder (FASD)	2	5.0%
Epilepsy	1	2.5%
Number of NDD diagnoses		
1	27	67.5%
>1	13	32.5%
Difficulty with daily activities		
Little difficulty	11	27.5%
Moderate difficulty	20	50.0%
A lot of difficulty	9	22.5%
Previous diagnosis of a mental health condition		
Yes	34	85.0%
No	6	15.0%
Current level of pain		
No pain	9	22.5%
Low pain	18	45.0%
Moderate pain	3	7.5%
A lot of pain	7	17.5%

Participant responses for disability questions were excluded in this table if the participant did not select a response or if they selected “prefer not to answer” or “do not know.” The number of respondents for some characteristics may not equal the total number of participants, and percentages may not add up to 100 percent.

^*^For the purposes of Table 2, we used a set of rules [outlined in a separate publication (34)] to ensure one primary diagnosis for participants that reported more than one NDD diagnosis.

### 3.2 Interview findings

Findings from the interviews provided insight into the ways in which the COVID-19 pandemic impacted and changed the lives of participants. Below, we present the core themes that emerged during analysis, which reflect impacts on the following: education and academic performance, access to disability and healthcare services, social connectedness, participation in society, and mental health. We also present a key secondary finding that emerged that was outside of the scope of our initial research question: gender plays an important role in the ability to access supports and services.

#### 3.2.1 Impacts on education and academic performance

One of the strongest themes that emerged in our analysis was the impact of the COVID-19 pandemic on the education and academic performance of participants. Key sub-themes that emerged relating to education included lack of accommodations and support, lack of flexibility in delivery, and considerations relating to online versus in-person delivery.

Participants experienced an overall lack of accommodations and support that met their specific learning needs, particularly during lockdown periods of the COVID-19 pandemic. In-person services, such as mental health support, inclusive education resources, and therapy, previously provided in school or on-campus were often not provided to the same extent in the virtual school setting. This resulted in challenges with learning, decreased support, negative impacts on academic performance, and increased anxiety. This is exemplified by one participant who expressed that, “And academically as well, it’s very difficult to receive the support that I need going into university. Communicating with the Disabled Students Association online is very, very, very difficult (Participant Y282).” Accommodations were also not readily adopted in educational settings, as discussed by one participant:

And it just, in that way, when I'm so self-conscious, I guess, [because] of my anxiety disorders and stuff, so I was not turning the camera on. But I had one instructor that I had to turn it on for some of [the class]… [I was] frustrated that such a simple accommodation couldn't be made. –Participant Y72

Additionally, many participants shared negative implications related to a lack of flexibility in education delivery during the different phases of the COVID-19 pandemic. As summarized by one participant in August 2021:

with the reopening of the world, the university that I attend is insisting that it's in-person only, they're not going to be offering hybrid, which is leaving me in a position of not being sure how to pursue my education and finish my degree because I am immunocompromised and can't risk being on campus, or in a class of 200 students. –Participant Y348

Participants’ perceptions of the effectiveness of and their satisfaction with online learning was mixed. Many shared that online learning was not an ideal option for them, as they felt that they needed in-person interaction to successfully learn class materials. Many found that access to their teachers and professors decreased in the virtual space, resulting in less support with class material and a heavier workload. This led to increased feelings of stress and burnout. Some participants found it difficult to stay on top of schoolwork while also dealing with the mental health impacts associated with the COVID-19 pandemic. One participant summarized this:

I would've liked more understanding about grades and assignments from my university - the teachers usually tried their best to be accommodating, but it seemed that the university began to 'crack down' on 'lenient grading' about halfway through the pandemic. I have pretty much constant brain fog, anxiety, and lack of access to basic self-care needs, and I feel like I'm being expected to function at normal levels by pretending the pandemic's not happening. –Participant Y196

Some participants preferred online learning for many reasons, which included the ability to pause classes and revisit material, decreased time commuting to and from campus, reduced exposure to inaccessible spaces (for example classrooms with bright florescent lighting, crowded and loud public spaces), and decreased social pressure. As shared by one participant:

For me, at least, the asynchronous classes were better for ADHD. And mostly because I had the ability to pause […] And I'm thinking now that having the recording, it doesn't really give anybody an advantage, other than you can hear the information again. You still need the ability to retain the information and to have it in your brain during a test and things like this. But people who struggle with focusing attention or just ADHD, and other learning disabilities in the classroom, it's a huge advantage to be able to, not even an advantage, but more of putting people into an equitable situation in the classroom. –Participant Y246

#### 3.2.2 Impacts on access to disability and healthcare services

Access to disability and healthcare services was another predominant theme of our findings, with access improving in some cases and worsening in others. Most participants reported that access to services was inconsistent throughout the pandemic; most services were completely cancelled during lockdown periods and virtual service options were not always provided. These changes had significant impacts on the mental health of participants.

The level of satisfaction with virtual services was mixed among participants. Some participants found virtual services helpful because they were more easily able to have a support person join their appointments, as described by one participant:

So on the phone works best, because then I can have my support person, who may not be available, or may not be in town, even, available to participate, because it is just online or on the phone or something like that. –Participant Y348

Other benefits mentioned by participants included the ability to attend virtual appointments in the comfort and safety of their homes and to have social connection during an otherwise isolating time.

Other participants found virtual services inaccessible because of, for example, challenges with lip reading and picking up on social and facial cues in a virtual space. Others found virtual services to be less effective due to the need to express needs and progress towards developmental and physical goals verbally, without an in-person, hands-on assessment. Participants residing in shared living spaces also found that they had a lack of privacy for virtual appointments. This is exemplified by the following quote:

I just find it really hard to focus on therapy when it's online, I'm getting distracted all the time and plus the privacy part was just really annoying that I didn't have a lot of privacy and I was worried about other people overhearing my therapy sessions. –Participant Y140

Many participants also discussed their experiences when accessing healthcare services during the COVID-19 pandemic. Many noted that long waitlists, especially for specialists, and the necessity in many cases to have a phone appointment prior to an in-person appointment resulted in delays in accessing medical care. Additionally, the inability to have a support worker or advocate come to in-person appointments due to visitation policies in hospitals and other medical settings was challenging for many participants. This difficulty was described by one participant:

Due to disability, I actually can't be at hospital by myself, it's a big safety risk. So it was an ongoing battle for every test, in order to get my support person approved to come in. The hospital policies were that nobody comes in under any circumstances, and because I'm over the age of 18, there wasn't the option to bring a parent or guardian, not that I have either of those. So with that, it was just an ongoing battle with the hospital. –Participant Y348

Despite these challenges, some participants shared positive, inclusive experiences accessing healthcare services during the pandemic, specifically during vaccine distribution. Many participants had no issue bringing their support person to their vaccine appointment. One participant shared their story of several accommodations that were made during their vaccine appointment:

I do have a severe fear of needles and things like that. So I do at times go non-verbal. So they were more than happy to accept my written note, that I had pre-written, of, "Yes. I consent to this vaccine," because typically you have to do all that stuff, too. They were more than happy to accept that, very accommodating. And even, because of medical conditions and allergies, I had to wait for 45 minutes in the space before I was allowed to leave, but they were more than happy to accommodate me and my worker going back out to the car. She just had to pull the car up within eyesight of the door … we actually had a public health nurse come out to us and book my second vaccine at the end of the 45-minute mark, when I was allowed to leave. –Participant Y89

Among participants, the two most expressed unmet needs with respect to service access during the pandemic were the ability to have access to COVID-safe, in-person social opportunities, such as social support groups or day programs, and consistent access to in-person support workers. Being able to access safe, in-person activities with others was very beneficial to the overall health and wellbeing of many participants, and many wished they had more opportunities for this throughout the pandemic. This is noted by one participant, who shared, “I would have liked social support groups - like many others I’ve felt very isolated during the pandemic (Participant Y196).”

One participant summarized the positive and negative impacts associated with changes in access to services during the COVID-19 pandemic:

I believe the increased prevalence of online services has had the beneficial effect of allowing people with autism spectrum disorder to attend appointments without having to leave their house, but on the flip side, the lack of in person appointments and overall backlog has definitely slowed down and reduced the effectiveness of care received. –Participant Y148

#### 3.2.3 Impacts on social connectedness

Participants experienced a deconstruction of their social network during the COVID-19 pandemic, which had both positive and negative impacts. For many participants, isolation from family and friends and a lack of social opportunities was challenging and resulted in mental health challenges such as depression, irritation, loneliness, stress, and anxiety. One participant describes this experience:

I think just me being a very social-oriented person, and that bothered me, feeling so isolated. And then not being able to go see my family played a big role in that. So I definitely had a harder time when restrictions were very limiting. –Participant Y122

Many felt that they had lost their social support system, which they expressed was essential to their overall health and wellbeing. Additionally, some participants noted that during re-opening periods when stay-at-home orders were relaxed, their levels of social anxiety were higher and they found it harder to socialize because of months of social isolation. As one participant shared, “I was isolated from my partner for several months, I feel extremely disconnected from other LGBTQ+ autistic people and communities. I think my social skills have declined drastically (Participant Y269).”

Alternatively, participants with sensory issues or social anxiety found isolation to be beneficial, as one participant describes: “And then I guess just not having to interact with people that much was really good for me and my mental health and my sensory needs (Participant Y140).” Because of the risk associated with social gatherings during the COVID-19 pandemic, participants found others were more accepting of their choice not to attend these gatherings. In turn, this improved their stress and lessened their social anxiety.

#### 3.2.4 Impacts on participation in society

As a result of precautionary health measures implemented during the COVID-19 pandemic, many participants experienced changes in their ability to interact with and access spaces and places associated with their regular, day-to-day activities. For some individuals, mask-wearing policies created challenges entering public spaces. Participants with sensory issues struggled to wear masks in public, as shared by one participant: “With my autism and sensory issues uh I cannot, it was hard wearing a mask, and with my Tourette’s, that was hard wearing a mask too (Participant Y249).” Some participants found it hard to assess social cues in public spaces with everyone in masks. This is described by one participant:

Being autistic, it's hard enough to read body language as is, and facial expressions, so I was just learning to do that, and then masks came around. It's like, ah crap. I can't tell what anybody's feeling at all. What do they mean by what they're saying? –Participant Y89

Participants with a mask-wearing exemption felt hesitant to enter public spaces without a mask because of fears that others would judge them. In combination, these experiences made many participants less inclined to enter public spaces, often confining them to their homes and isolating them from society. This experience is exemplified by one participant, “And sometimes I’ll even just get handed a mask, and asked to put this on, so then that makes it even more awkward for me, so I just leave (Participant Y122).” Alternatively, for some participants mask wearing improved confidence and decreased the need for them to control their facial expressions in public. One participant shared:

I love wearing masks. We don't have a mask mandate where I live anymore, but I have not stopped because I genuinely love wearing masks. It makes me feel more anonymous and gives me confidence because I feel like I'm being stared at less. It's comforting. – Participant Y196

Additionally, some participants noted positive impacts on the physical landscape of public spaces. These participants found that directional arrows and social distancing floor markers provided a clear set of rules for existing in public spaces, leading to decreased anxiety and increased comfort being in public spaces. One participant noted:

Having clear guidelines has made it easier for me to navigate the world. In stores, there are instructions of which direction to walk, which lowers my anxiety as I then know what is expected of me. Keeping six feet apart prevents me from becoming crowded and stressed. Frequent handwashing means that there is sanitizer present at every doorway in a public space. –Participant Y311

#### 3.2.5 Impacts on mental health

The vast majority of participants experienced changes to their mental health during the COVID-19 pandemic. The most common mental health changes experienced by participants were feeling stressed or worried, mood changes, irritability, and feeling lonely/isolated. Participants attributed these mental health challenges primarily to changes in service access and education, social isolation, and implementation of precautionary health measures, which are explored in detail in the previous sections.

The different phases of the COVID-19 pandemic had differing impacts on the mental health of participants, with the majority noting that they experienced changing mental health as the pandemic progressed through different phases. Some participants found that their mental health improved during re-opening periods of the pandemic, due to increased opportunities for in-person social interaction and increased ability to access services. As shared by one participant, “Well during the lockdown, it was very difficult. I was going stir crazy. I was restless, depressed, anxious. And then when things started [to] open up again, I started to feel a bit better (Participant Y422).” For others, re-opening periods increased feelings of stress and anxiety due to the increased risk of contracting COVID-19 and the need to return to a higher level of day-to-day social interactions. This is exemplified by one participant, who said, “When we are in lockdown, I feel a lot safer because people are staying home and staying away from each other. But when we are not in lockdown and there’s not many vaccines out there, it’s kind of scary (Participant Y84).”

When asked how participants coped with changes to their mental health during the COVID-19 pandemic, many participants shared that they accessed mental health support from a professional, with some accessing support for the first time and others relying on support they already had in place. Of those that accessed professional mental health support during the pandemic, many highlighted barriers to accessing consistent and accessible support. Many noted that long waitlists for mental health practitioners and limited available appointments made it difficult to get timely support. One participant exemplifies this by sharing, “I relied on crisis lines for support because of the lack of ability to access service (Participant Y165).” Further, some participants seeking support for the first time discovered that their options for accessible and inclusive mental health support were limited, as described by one participant:

I've been wanting to start therapy for a while now, but it was inaccessible due to the fact that I couldn't find a therapist accepting in person appointments. Since I'm autistic, I struggle a lot with auditory processing which makes it difficult to have conversations over the phone or on zoom. Therapy would have become another source of stress in my life because of this. – Participant Y374

#### 3.2.6 Secondary finding: impact of gender on access to services

When asked if gender had an impact on the experiences of youth during COVID, many participants shared their experiences with gender and service access outside the specific context of the COVID-19 pandemic, but as a factor impacting their access to different services and opportunities. Considering the fact that the majority of participants indicated that they think that gender (or perception of their gender) influenced the type of services provided to them and how they were treated by service providers, we include a high level overview here as a key secondary finding of this study. We highlight this as an important topic for future exploration.

##### 3.2.6.1 Diagnostic biases

Many participants shared that in their view, there is a lack of understanding among medical professionals and service providers regarding the differences in disability presentation among women and girls. Many shared that they experienced delays in diagnosis or accessing support because they did not conform to the stereotypical presentation of their disability, which is often based on males. This is exemplified by the following quote from a participant:

I've had service providers disbelieve my autism diagnosis because I'm a woman and chalk autistic behaviours up to a mental health or personality disorder instead. I do have comorbid mental health diagnoses in addition, but some service providers have suggested that some of my autistic traits are me being willfully difficult or manipulative. – Participant Y196

##### 3.2.6.2 Barriers to accessing support

Many participants felt that gender-related stereotypes impeded their ability to receive appropriate supports. Some participants discussed that they found services to be targeted primarily to cisgender men, making it hard for individuals with other gender identities to get access to the support they need in a safe space. One participant noted:

I'm an autistic cis woman, and I find that autism services as an adult (as it was as a teen) is mainly geared towards cisgender men. Social support groups are comprised of men. Skills coaching in a group is geared towards men, and taught by men. It makes me feel even more alienated than I already do. – Participant Y169

Alternatively, some male-identifying individuals expressed their difficulties in accessing needed mental health services, which they attributed to the stigma associated with males seeking support for their mental health. This is described by one participant:

The ways that shame and difficulty with asking for help is even more challenging amongst men/certain masculine people, leading to getting less or no services when needed. [There are] assumptions about what one gender might need help with more than another. – Participant Y136

##### 3.2.6.3 Safe spaces

Finally, the non-binary, transgender, and gender non-conforming individuals that we interviewed often found it difficult to find safe spaces where their gender identity was respected. Many participants shared that they hesitated to access services due to fears of discrimination, transphobia, or misgendering. Participants also shared that service providers were not always educated about differing gender identities, resulting in a failure to respect their gender identity and pronouns. One participant summarized this:

As someone who identifies as genderfluid, I have found that many employers or service providers refuse to respect my pronouns, make assumptions that my gender identity is a mental illness from my disability and are very dismissive and degrading of it. – Participant Y306

## 4 Discussion

This study used a qualitative design to understand the experiences of Canadian youth with NDD during the COVID-19 pandemic. Our analysis highlighted both the challenges and, for some, benefits of precautionary health policies implemented during the pandemic. Our analysis also provides insight into the impact of gender on the ability of participants to access services and supports—an area that warranted mention but requires future exploration for greater depth. The results highlight the unique and varied needs among a diverse group of youth with various NDDs during the COVID-19 pandemic. To our knowledge, this is the first study investigating the impacts of the COVID-19 pandemic from the perspective of youth with NDD, themselves. We also utilize a novel approach to interviewing with a written and verbal option, which enhanced the accessibility of our study.

We highlight the impacts of the COVID-19 pandemic on Canadian youth with NDD in five key areas: education and academic performance, access to disability and healthcare services, social connectedness, participation in society, and mental health. Across these areas, we describe the unintended positive and negative impacts of the COVID-19 pandemic on the lives of youth with NDD and their families. This demonstrates the diverse and varying experiences among youth with NDD and their families, and the need to consider and account for diverse needs in the development of pandemic-related policy. Our results also show that a one-size fits all and inflexible approach, like the one used during the pandemic to design and implement pandemic-related policy, had the consequence, in some cases, of leaving individuals with disabilities, an already vulnerable group, even further behind. While the rushed implementation of pandemic-related policy was necessary to contain the impacts of the COVID-19 pandemic and protect the health and safety of the population, it had the inadvertent consequence of impacting vulnerable groups, including those with disabilities.

As a signatory to the United Nations Convention on the Rights of Persons with Disabilities (UN CRPD), Canada is obligated to take “all necessary measures to ensure the protection and safety of persons with disabilities in situations of risk, including situations of armed conflict, humanitarian emergencies and the occurrence of natural disasters,” as stated in Article 11 (26). The one-size fits all policies implemented during the COVID-19 pandemic across Canada did not fully address this obligation, as they fell short in consistently and fully considering the unique and variable needs of youth with NDD, and individuals with other disabilities more broadly. This notion is confirmed by a quantitative analysis highlighting the lack of engagement of children and youth with disabilities, their families, and/or representative organizations to develop disability-inclusive policies during the COVID-19 pandemic (27). This work also noted that, while pandemic policy documents often mentioned the challenges faced by youth with disabilities and their families during the pandemic, few offered mitigation strategies for these challenges and considered protection of essential human rights through the course of the pandemic (27).

The shortcomings of the one-size fits all approach implemented during the COVID-19 pandemic, as evidenced by our findings and the findings of others, can be viewed as a case study that offers an important lesson about the need to move towards designing and implementing policy that is disability-inclusive. This is not only important in the context of COVID-19 recovery efforts and future emergency planning (28–30), but also beyond the pandemic to ensure that policy implemented across sectors is inclusive of the needs of individuals with disabilities and their families. This is in alignment with the Government of Canada’s *Disability Inclusion Action Plan,* which outlines a whole-of-government approach to disability inclusion guided by the following three principles: (1) “Nothing Without Us,” which outlines the need for persons with disabilities to be involved in the development and implementation of all government systems, policies, programs, and services; (2) Human-rights based approach, which outlines the need to include principles of equality, anti-discrimination, participation, and inclusion in the process of developing systems and processes; and, (3) Intersectionality, which outlines the need to take into account the multiple, intersecting forms of marginalization and discrimination faced by some individuals with disabilities (31).

Critical to achieving disability-inclusion across policy and practice is the use of a co-design approach. Co-design is an inclusive and collaborative process that must include a diverse range of individuals with relevant skills, lived experience, or expertise, and ensures that relevant stakeholders are at the center of decision-making throughout the design process (32, 33). Co-design involves the recognition that people that are impacted by policy have valuable expertise and perspectives that can and should be integrated into the policy process. In the context of disability-inclusive policy, stakeholders that should be included in co-design are individuals with lived experience, parents and family members, formal and informal caregivers, and representative organizations, such as non-profits and service provision organizations. In line with principles outlined in the *Disability Inclusion Action Plan* above and with our preliminary results regarding the impact of differing gender identities on ability to access services, there is a need to ensure a diverse group of individuals are included in the co-design process, spanning different genders, income levels, community types, diagnoses, ages, races, ethnicities, and other identity and social positioning attributes. A co-design approach to policy design and implementation is essential to mitigate the unintended impacts of policy changes to avoid perpetuating further harm against the disability community and ensure that policy is equitable and inclusive of the needs of all individuals with disabilities.

### 4.1 Strengths and limitations

Our study has several strengths. First, we provide an in-depth overview of the experiences of forty youth with NDD during the COVID-19 pandemic from the perspectives of youth, themselves, and not a parent or caregiver. Second, having two options for completing the interview enhanced the accessibility of the study for youth, and allowed us to capture the perspectives of individuals that we may not have otherwise been able to reach.

Despite the strengths noted above, there are limitations to our study that warrant mention. Our sample had a lack of representation of individuals residing in northern Canada, i.e., Northwest Territories, Yukon Territory, Nunavut, and in two Atlantic provinces: Newfoundland and Labrador and Prince Edward Island. Our sample also had a small proportion of individuals self-identifying as Indigenous (although a larger proportion relative to the general population) and of French-speaking individuals. Understanding the specific challenges experienced by Indigenous peoples and French Canadians with NDD is an important area of future research and would help strengthen the ability to conduct an in-depth intersectional analysis. Second, our reliance on the internet to access both forms of the interview means we likely did not capture the voices of those living in more rural and remote areas that may have limited access to the internet. Third, there is potential for self-selection bias among individuals that participated in the study as a result of being connected with the research team or advisory council. However, we do feel that this potential for bias was balanced by our social media recruitment (particularly our use of social media ads). Fourth, there is an overrepresentation of autism spectrum disorder among our participants, which may limit generalizability to all youth with disabilities. Finally, due to the reliance on participant recall of experiences, there may have been recall bias or memory distortion during interviews.

## 5 Conclusion

In this study, we uncovered the experiences of youth with NDD during the COVID-19 pandemic across Canada. The impacts of the COVID-19 pandemic on youth with NDD revealed through this study underscore the importance of fully considering the needs of youth with NDD and their families when designing and implementing policy. To achieve this, we recommend governments and other decision-makers consider applying a co-design approach to policy design and implementation whenever possible. While our research is focused on experiences in both the Canadian context and the context of the COVID-19 pandemic, we believe interpretations of our results are applicable across sectors and jurisdictions.

Future research should build on this work to determine policy options that are more disability-inclusive, to ensure youth with NDD and their families are adequately supported across their lifespan, in both emergency and non-emergency situations. This should involve engaging youth with NDD and other relevant stakeholders directly, through an inclusive co-design approach.

The COVID-19 pandemic, while a difficult time for many across the world, provides a valuable learning opportunity for decision-making entities with respect to the need to implement disability-inclusive policy moving forward. We hope that our work contributes to an enhanced understanding of the consequences of implementing non-disability-inclusive policy and encourages more thoughtful and inclusive policy development in the future.

## Data Availability

The raw data supporting the conclusions of this article will be made available by the authors, without undue reservation.
